# Sociodemographic and psychological study on performance of students for the COVID-19 aftermath dataset

**DOI:** 10.1016/j.dib.2020.106421

**Published:** 2020-10-17

**Authors:** Siti Feirusz Ahmad Fesol, Mohd Mursyid Arshad

**Affiliations:** a Faculty of Computer Science and Mathematics, Universiti Teknologi MARA (Melaka), Jasin Campus, Melaka, Malaysia; b Faculty of Educational Studies, Universiti Putra Malaysia, Serdang, Selangor, Malaysia

**Keywords:** Learning habits, Sociodemographic, Psychological, Online learning, COVID-19

## Abstract

This paper presents the dataset of undergraduates learning habits during and before the occurrence of pandemic COVID-19 under the scope of sociodemographic and psychological aspects. This dataset consists of four (4) main sections which are students' demographic, psychological disruption, students' learning habits and integration of online sessions with sustainability topics. A total of 37 variables were distributed via an online survey platform. The link of the online survey was circulated to the students using few social media platforms such as WhatsApp groups, Telegram, and faculties' Facebook starting from June 1 until June 31, 2020. There was a total of 668 respondents accompanied by consent were agreed to join the survey. This dataset can have an important role for research and education in identifying the impact on learning performance among the undergraduate students during COVID-19 pandemic based on different sociodemographic and psychological aspects.

## Specifications Table

**Table:** 

Subject	Education
Specific subject area	Learning habits, Online learning, Sociodemographic, Psychological
Type of data	Table
How data were acquired	Online survey
	Link: https://forms.gle/Mhcm6xRvjpGDym327
Data format	Raw
	Analyzed
Parameters for data collection	The target respondents of this survey were undergraduate students from public university in Malaysia, across different faculties, who are learning effected due to COVID-19.
Description of data collection	The survey form was distributed via an online platform. The link of the online survey was circulated to the students using few social media platforms such as WhatsApp groups, Telegram, and faculties' Facebook.
Data source location	Institution: Universiti Teknologi MARA
	Region: Asia
	Country: Malaysia
Data accessibility	Repository name: Mendeley repository
	Direct URL to data: http://dx.doi.org/10.17632/dspbfsp9ds.3

## Value of the Data

• The dataset covered information of students' learning habits before and during COVID-19.
• Useful dataset for researchers who interested to identify effects and analyze the impact of students' learning habits during COVID-19 among different sociodemographic status.
• The dataset can be served as a reference source for researchers who interested to identify the relationship between psychological disruption impact on students' necessity of self-learning and self-motivation towards effective learning during COVID-19.
• The dataset is a reference source and guideline for policy makers in enhancing the future policies with regards to the online learning which can be aligned with the students' different sociodemographic and psychological aspects as well as betterment of education systems preparation for similar situations in the future.

## 1 Data Description

The landscape of education sector around the world has drastically changed due to the spread of the Novel Corona Virus Disease 2019 or Covid-19 [1]. Thus, online digital learning has taken place to support the continuation of teaching and learning process during the pandemic, which has eventually impacted the students’ learning habits [2, 3]. In response to this, this dataset [4] describes undergraduates learning habits before and during the occurrence of COVID-19 pandemic and its mediating factors, which include the learning hours, different socioeconomic status, students' perception of psychological disruption, students’ perception of the necessity of self-learning and the self-motivation factors that support students’ effective learning. The target respondents of this survey [4] were undergraduate students from a public university in Malaysia, across different faculties, who are their learning affected due to COVID-19. Table 1 shows the descriptive statistics of students' demographics. The demographics items consist of gender, current year of study, level of study, reside area, occupation sector of head of family, occupation field of the head of family, and total family income per month. The minimum and maximum column reflected as the minimum and maximum value answered by the user for each demographic's items.

**Table 1:** Descriptive statistics of students' demographics.

		Frequency	Percent	Minimum	Maximum
Gender	Male	299	44.8	1	2
	Female	369	55.2	1	2
	Total	668	100.0		
Current year of study	1st & 2nd year	436	65.3	1	4
	3rd & 4th year	232	34.7	1	4
	Total	668	100.0		
Level of study	Diploma	265	39.7	1	2
	Degree	403	60.3	1	2
	Total	668	100.0		
Reside area	Rural area (Countryside)	275	41.2	1	2
	Urban area (Town/City)	393	58.8	1	2
	Total	668	100.0		
Occupation sector of head of family	Government sector	212	31.7	1	5
	Private sector	195	29.2	1	5
	Self-employed	146	21.9	1	5
	Unemployed	71	10.6	1	5
	Others	44	6.6	1	5
	Total	668	100.0		
Occupation field of the head of family	Manager and Professional	99	14.8	1	8
	Technical and Associate Professionals	97	14.5	1	8
	Clerical Support Workers	57	8.5	1	8
	Service and Sales Workers	96	14.4	1	8
	Skilled Agricultural, Forestry, Livestock and Fisheries Workers	36	5.4	1	8
	Craft and Related Trades Workers	11	1.6	1	8
	Plant and Machine Operators and Assemblers	24	3.6	1	8
	Other	248	37.1	1	8
	Total	668	100.0		
Total family income per month (RM)	Less than RM4000	346	51.8	1	3
	RM4000 - RM9000	222	33.2	1	3
	More than RM9000	100	15.0	1	3
	Total	668	100.0		

Table 2 summarizes a cross tabulation results between students' demographics and learning habits measure by learning hours each student used per day before and during the pandemic COVID-19. The learning hours were categorized into three (3) groups which are less than 4 h per day, 4–8 h per day, and more than 8 h per day.

**Table 2:** Crosstab results between students' demographics and learning habits (hours per day).

			Learning hours before COVID-19				Learning hours during COVID-19			
	Variables		< 4	4–8	> 8	Total	< 4	4–8	> 8	Total
Gender	Male	Count	206	90	3	299	180	113	6	299
		% within gender	68.9%	30.1%	1.0%	100.0%	60.2%	37.8%	2.0%	100.0%
		% within lh_before	48.9%	39.6%	15.0%	44.8%	53.9%	39.4%	12.8%	44.8%
		% of Total	30.8%	13.5%	.4%	44.8%	26.9%	16.9%	.9%	44.8%
	Female	Count	215	137	17	369	154	174	41	369
		% within gender	58.3%	37.1%	4.6%	100.0%	41.7%	47.2%	11.1%	100.0%
		% within lh_before	51.1%	60.4%	85.0%	55.2%	46.1%	60.6%	87.2%	55.2%
		% of Total	32.2%	20.5%	2.5%	55.2%	23.1%	26.0%	6.1%	55.2%
Current year of study	1st & 2nd year	Count	273	151	12	436	226	175	35	436
		% within sem	62.6%	34.6%	2.8%	100.0%	0.52	40.1%	8.0%	100.0%
		% within lh_before	64.8%	66.5%	60.0%	65.3%	67.7%	61.0%	74.5%	65.3%
		% of Total	40.9%	22.6%	1.8%	65.3%	33.8%	26.2%	5.2%	65.3%
	3rd & 4th year	Count	148	76	8	232	108	112	12	232
		% within sem	63.8%	32.8%	3.4%	100.0%	46.6%	48.3%	5.2%	100.0%
		% within lh_before	35.2%	33.5%	40.0%	34.7%	32.3%	39.0%	25.5%	34.7%
		% of Total	22.2%	11.4%	1.2%	34.7%	16.2%	16.8%	1.8%	34.7%
Level of study	Diploma	Count	176	83	6	265	144	106	15	265
		% within edu_level	66.4%	31.3%	2.3%	100.0%	54.3%	40.0%	5.7%	100.0%
		% within lh_before	41.8%	36.6%	30.0%	39.7%	43.1%	36.9%	31.9%	39.7%
		% of Total	26.3%	12.4%	.9%	39.7%	21.6%	15.9%	2.2%	39.7%
	Degree	Count	245	144	14	403	190	181	32	403
		% within edu_level	60.8%	35.7%	3.5%	100.0%	47.1%	44.9%	7.9%	100.0%
		% within lh_before	58.2%	63.4%	70.0%	60.3%	56.9%	63.1%	68.1%	60.3%
		% of Total	36.7%	21.6%	2.1%	60.3%	28.4%	27.1%	4.8%	60.3%
Reside area	Rural area (Countryside)	Count	164	100	11	275	143	111	21	275
		% within reside_area	59.6%	36.4%	4.0%	100.0%	52.0%	40.4%	7.6%	100.0%
		% within lh_before	39.0%	44.1%	55.0%	41.2%	42.8%	38.7%	44.7%	41.2%
		% of Total	24.6%	15.0%	1.6%	41.2%	21.4%	16.6%	3.1%	41.2%
	Urban area (Town/City)	Count	257	127	9	393	191	176	26	393
		% within reside_area	65.4%	32.3%	2.3%	100.0%	48.6%	44.8%	6.6%	100.0%
		% within lh_before	61.0%	55.9%	45.0%	58.8%	57.2%	61.3%	55.3%	58.8%
		% of Total	38.5%	19.0%	1.3%	58.8%	28.6%	26.3%	3.9%	58.8%
Occupation sector of head of family	Government sector	Count	136	71	5	212	104	98	10	212
		% within occ_head	64.2%	33.5%	2.4%	100.0%	49.1%	46.2%	4.7%	100.0%
		% within lh_before	32.3%	31.3%	25.0%	31.7%	31.1%	34.1%	21.3%	31.7%
		% of Total	20.4%	10.6%	.7%	31.7%	15.6%	14.7%	1.5%	31.7%
	Private sector	Count	121	70	4	195	90	86	19	195
		% within occ_head	62.1%	35.9%	2.1%	100.0%	46.2%	44.1%	9.7%	100.0%
		% within lh_before	28.7%	30.8%	20.0%	29.2%	26.9%	30.0%	40.4%	29.2%
		% of Total	18.1%	10.5%	.6%	29.2%	13.5%	12.9%	2.8%	29.2%
	Self-employed	Count	87	55	4	146	74	58	14	146
		% within occ_head	59.6%	37.7%	2.7%	100.0%	50.7%	39.7%	9.6%	100.0%
		% within lh_before	20.7%	24.2%	20.0%	21.9%	22.2%	20.2%	29.8%	21.9%
		% of Total	13.0%	8.2%	.6%	21.9%	11.1%	8.7%	2.1%	21.9%
	Unemployed	Count	46	21	4	71	38	31	2	71
		% within occ_head	64.8%	29.6%	5.6%	100.0%	53.5%	43.7%	2.8%	100.0%
		% within lh_before	10.9%	9.3%	20.0%	10.6%	11.4%	10.8%	4.3%	10.6%
		% of Total	6.9%	3.1%	.6%	10.6%	5.7%	4.6%	.3%	10.6%
	Others	Count	31	10	3	44	28	14	2	44
		% within occ_head	70.5%	22.7%	6.8%	100.0%	63.6%	31.8%	4.5%	100.0%
		% within lh_before	7.4%	4.4%	15.0%	6.6%	8.4%	4.9%	4.3%	6.6%
		% of Total	4.6%	1.5%	.4%	6.6%	4.2%	2.1%	.3%	6.6%
Occupation field of the head of family	Manager and Professional	Count	62	34	3	99	46	42	11	99
		% within occ_field	62.6%	34.3%	3.0%	100.0%	46.5%	42.4%	11.1%	100.0%
		% within lh_before	14.8%	15.0%	15.0%	14.9%	13.9%	14.6%	23.4%	14.9%
		% of Total	9.3%	5.1%	.5%	14.9%	6.9%	6.3%	1.7%	14.9%
	Technical and Associate Professionals	Count	63	33	1	97	39	50	8	97
		% within occ_field	64.9%	34.0%	1.0%	100.0%	40.2%	51.5%	8.2%	100.0%
		% within lh_before	15.1%	14.5%	5.0%	14.6%	11.8%	17.4%	17.0%	14.6%
		% of Total	9.5%	5.0%	.2%	14.6%	5.9%	7.5%	1.2%	14.6%
	Clerical Support Workers	Count	32	24	0	56	35	19	2	56
		% within occ_field	57.1%	42.9%	.0%	100.0%	62.5%	33.9%	3.6%	100.0%
		% within lh_before	7.7%	10.6%	.0%	8.4%	10.6%	6.6%	4.3%	8.4%
		% of Total	4.8%	3.6%	.0%	8.4%	5.3%	2.9%	.3%	8.4%
	Service and Sales Workers	Count	64	28	4	96	47	44	5	96
		% within occ_field	66.7%	29.2%	4.2%	100.0%	49.0%	45.8%	5.2%	100.0%
		% within lh_before	15.3%	12.3%	20.0%	14.4%	14.2%	15.3%	10.6%	14.4%
		% of Total	9.6%	4.2%	.6%	14.4%	7.1%	6.6%	.8%	14.4%
	Skilled Agricultural, Forestry, Livestock and Fisheries Workers	Count	19	14	1	34	20	13	1	34
		% within occ_field	55.9%	41.2%	2.9%	100.0%	58.8%	38.2%	2.9%	100.0%
		% within lh_before	4.5%	6.2%	5.0%	5.1%	6.0%	4.5%	2.1%	5.1%
		% of Total	2.9%	2.1%	.2%	5.1%	3.0%	2.0%	.2%	5.1%
	Craft and Related Trades Workers	Count	6	4	1	11	5	5	1	11
		% within occ_field	54.5%	36.4%	9.1%	100.0%	45.5%	45.5%	9.1%	100.0%
		% within lh_before	1.4%	1.8%	5.0%	1.7%	1.5%	1.7%	2.1%	1.7%
		% of Total	.9%	.6%	.2%	1.7%	.8%	.8%	.2%	1.7%
	Plant and Machine Operators and Assemblers	Count	17	4	3	24	12	11	1	24
		% within occ_field	70.8%	16.7%	12.5%	100.0%	50.0%	45.8%	4.2%	100.0%
		% within lh_before	4.1%	1.8%	15.0%	3.6%	3.6%	3.8%	2.1%	3.6%
		% of Total	2.6%	.6%	.5%	3.6%	1.8%	1.7%	.2%	3.6%
	Other	Count	155	86	7	248	127	103	18	248
		% within occ_field	62.5%	34.7%	2.8%	100.0%	51.2%	41.5%	7.3%	100.0%
		% within lh_before	37.1%	37.9%	35.0%	37.3%	38.4%	35.9%	38.3%	37.3%
		% of Total	23.3%	12.9%	1.1%	37.3%	19.1%	15.5%	2.7%	37.3%
Total family income per month (RM)	Less than RM4000	Count	198	137	11	346	176	147	23	346
		% within income	57.2%	39.6%	3.2%	100.0%	50.9%	42.5%	6.6%	100.0%
		% within lh_before	47.0%	60.4%	55.0%	51.8%	52.7%	51.2%	48.9%	51.8%
		% of Total	29.6%	20.5%	1.6%	51.8%	26.3%	22.0%	3.4%	51.8%
	RM4000 - RM9000	Count	151	63	8	222	109	96	17	222
		% within income	68.0%	28.4%	3.6%	100.0%	49.1%	43.2%	7.7%	100.0%
		% within lh_before	35.9%	27.8%	40.0%	33.2%	32.6%	33.4%	36.2%	33.2%
		% of Total	22.6%	9.4%	1.2%	33.2%	16.3%	14.4%	2.5%	33.2%
	More than RM9000	Count	72	27	1	100	49	44	7	100
		% within income	72.0%	27.0%	1.0%	100.0%	49.0%	44.0%	7.0%	100.0%
		% within lh_before	17.1%	11.9%	5.0%	15.0%	14.7%	15.3%	14.9%	15.0%
		% of Total	10.8%	4.0%	.1%	15.0%	7.3%	6.6%	1.0%	15.0%

Next, Table 3 shows the descriptive results of psychological disruption faced by the students which measured by the students’ experienced on certain scenario which are their health care access, internet access, ability to pursue studies, ability to socialize, and their overall psychological wellbeing, including and/or depression.

**Table 3:** Descriptive statistics of psychological disruption.

	*N*	Range	Min	Max	Sum	Mean		Std. deviation
Variables	Statistic	Statistic	Statistic	Statistic	Statistic	Statistic	Std. error	
***During the last few months, have you experienced any of the following? (Yes = 1; No = 2)***								
Help or assistance from a stranger.	668	1	1	2	1178	1.763	0.016	0.425
Adverse discrimination from a stranger.	668	1	1	2	1259	1.885	0.012	0.320
Difficulties due to changes in your living conditions, including hostel disclosures.	668	1	1	2	940	1.407	0.019	0.492
Difficulties in traveling.	668	1	1	2	912	1.365	0.019	0.482
***Relative to BEFORE COVID-19 crisis, how would you rank your CURRENT level of:***^a^								
Health care access	668	5	0	5	1495	2.238	0.054	1.402
Internet access	668	5	0	5	1494	2.237	0.047	1.214
Ability to pursue your studies, including your graduation and/or degree completion	668	5	0	5	1245	1.864	0.043	1.120
Ability to socialize	668	5	0	5	1305	1.954	0.044	1.129
Overall psychological wellbeing, including and/or depression	668	5	0	5	1243	1.861	0.044	1.150

a Rating scale: 0 = *N*/A or Don't Know; 1=Much worse than before; 2=Worse than before; 3=Same as before; 4=Better than before; 5=Much better than before.

Following, Table 4 shares on the students' perception on self-learning which measured by necessity towards the self-learning during COVID-19 and self-learning effectives aspect.

**Table 4:** Descriptive statistics of students' perception on self-learning.

Variables	*N*	Range	Min	Max	Sum	Mean		Std. deviation
	Statistic	Statistic	Statistic	Statistic	Statistic	Statistic	Std. error	
***I think that self-learning during COVID-19 is necessary because:***^a^								
I can assure my learning progress	668	4	1	5	2073	3.103	0.041	1.062
I can maintain my learning habit	668	4	1	5	1951	2.921	0.044	1.125
My lecturers advise/inform me it is necessary and important.	668	4	1	5	2351	3.519	0.036	0.919
My parents advise/inform me it is necessary and important.	668	4	1	5	2230	3.338	0.039	0.997
My siblings advise/inform me it is necessary and important.	668	4	1	5	2096	3.138	0.038	0.984
My friends advise/inform me it is necessary and important.	668	4	1	5	2208	3.305	0.039	1.014
***I consider my self-learning activities are effective because: ^a^***								
I have motivation for self-learning	668	4	1	5	1790	2.680	0.042	1.098
I have proper concentration skill	668	4	1	5	1726	2.584	0.040	1.045
I can define my daily learning objectives	668	4	1	5	1835	2.747	0.038	0.993
I have support from my family	668	4	1	5	2183	3.268	0.039	1.019
I have an effective learning environment	668	4	1	5	1815	2.717	0.043	1.108
I have various learning resources	668	4	1	5	2049	3.067	0.042	1.074
I communicate and collaborate with my friends about learning	668	4	1	5	2136	3.198	0.042	1.081

a Rating scale: 1=Strongly disagree; 2=Disagree; 3=Neither agree nor disagree; 4=Agree; 5=Strongly agree.

While Table 5 summarizes the descriptive statistics of students' perception on online sessions with regards to the sustainability topics such as preventive health care, Coronavirus, sustainable environment development, and E-learning tools and techniques. Detailed descriptions of all the variables and questions used for this study can be found in the Mendeley data repository [4]. The complete survey form can be found in the supplementary file.

**Table 5:** Descriptive statistics of students' perception on online sessions with sustainability topics.

Variables	*N*	Range	Min	Max	Sum	Mean		Std. deviation
	Statistic	Statistic	Statistic	Statistic	Statistic	Statistic	Std. error	
***During COVID-19 crisis, I have learnt additional knowledge on: ^2^***								
Preventive health care	668	4	1	5	2599	3.891	0.031	0.813
Coronavirus	668	4	1	5	2678	4.009	0.031	0.799
Sustainable environment development	668	4	1	5	2513	3.762	0.033	0.842
E-learning tools and techniques	668	4	1	5	2550	3.817	0.032	0.828

## 2 Experimental Design, Materials and Methods

This dataset [4] consist of four (4) main sections which are Section A related to students' demographic, Section B related to psychological disruption, Section C related to students' learning habits, and Section D related to integration of online sessions with sustainability topics adopted from [5] and [6]. A survey form consist of 37 items were distributed via an online survey. The link of the online survey was circulated to the students from the respective lecturers using few social media platforms. Such as WhatsApp groups, Telegram groups, and faculties' Facebook starting from June 1 until June 31, 2020. There was a total of 674 feedback was collected however, 6 of them are refused to join the survey. The remaining 668 respondents accompanied by consent were agreed to join the survey.

The data were first gone through a data cleaning process to identify missing values and performed corrective action with regards to it. Next, the data were analyze using frequency analysis (see Table 1). For the purpose to analyze the difference in students’ learning habits before and during pandemic COVID-19, a cross tabulation analysis was conducted between students' demographics variables and learning habits variables (see Table 2).

A summary statistic for students' perception on the level of their psychological disruption, the necessity towards self-learning, and additional knowledge with regards to sustainability topics during COVID-19 datasets are presented in Table 3-5. These statistics were obtained using descriptive analysis as suggested by Trung et al. [5].

## Ethics Statement

An informed consent was obtained for experimentation with human subjects. All the respondents were asked for their consent before they can answer the survey.

## Declaration of Competing Interest

The authors declare that they have no known competing financial interests or personal relationships that could have appeared to influence the work reported in this paper.

## Appendix Supplementary Materials

Image, application 1
