# Supplementary material for: Sociodemographic and psychological study on performance of students for the COVID-19 aftermath dataset
Source: Data Brief. 2020 Oct 17;33:106421. doi: 10.1016/j.dib.2020.106421 (PMC7567699; doi:10.1016/j.dib.2020.106421)
Supplement: Supplementary file 1 [file mmc1.pdf]

Survey on Student's Learning Behaviour Amid COVID-19 Pandemic

You are invited to participate in a research study. This survey will take between 10 and 15 minutes to complete. Every question is entirely voluntary. The survey does not collect any personally identifiable information, and your responses will always remain strictly anonymous.

This survey is conducted in relation to identify the students' learning behaviour during this COVID-19 pandemic and the level of difficulties that they experienced. It will serve these purposes best if items are answered carefully and honestly as you reflect back on your learning experience through this pandemic period. All the information gathered will be used for the academic purpose only.

If you have any questions or comments, please do not hesitate to contact us via email at: nurulhudaghazali@uitm.edu.my

The survey display is optimized for desktop browser with keyboard input. If you are on a mobile phone, you may need to rotate your display to respond to some questions.
* Required

1. To start the survey, please select a response below : *

Mark only one oval.

- ( ) I would like to participate in the survey AND I am 18 years or older
- ( ) I would NOT like to participate the survey AND/OR I am not 18 years or older
  Skip to section 5 (Your cooperation and honesty for taking part in this survey are highly appreciated. Thank you.)

Section A.

This section will gather your demographic information.

2. What is your current age? *

Mark only one oval.

- Less than 18
- 18-21
- 22-25
- 26-29
- More than 29

3. What is your gender? *

Mark only one oval.

- Male
- Female

4. Which campus are you currently pursuing your study? *

Mark only one oval.

- Jasin
- Alor Gajah
- Bandaraya Melaka

5. Which faculty are you currently pursuing your study? *

Mark only one oval.

- Faculty of Computer & Mathematical Sciences
- Faculty of Plantation & Agrotechnology
- Faculty of Communication & Media Studies
- Faculty of Art & Design
- Faculty of Accountancy
- Faculty of Business & Management
- Faculty of Hotel & Tourism Management

6. Which semester are you in? *

Mark only one oval.

- 1st Semester
- 2nd Semester
- 3rd Semester
- 4th Semester
- 5th Semester
- 6th Semester
- 7th Semester
- 8th Semester
- 9th Semester
- 10th Semester
- None of the above

7. What level of education are you currently pursuing? *

Mark only one oval.

- Diploma
- Undergraduate (Bachelor)
- Master
- PhD or Postdoctoral

8. Has your institution moved to virtual instruction due to the COVID-19 crisis? *

Mark only one oval.

- Yes
- No

9. Where do you reside (live) currently? *

Mark only one oval.

- Rural area (Countryside)
- Urban area (Town/City)

10. Occupation sector of the head of family : *

Mark only one oval.

- Government sector
- Private sector
- Self-employed
- Unemployed
- Other: ____________________

11. Occupation field of the head of family : *

Mark only one oval.

- Manager and Professional
- Technical and Associate Professionals
- Clerical Support Workers
- Service and Sales Workers
- Skilled Agricultural, Forestry, Livestock and Fisheries Workers
- Craft and Related Trades Workers
- Plant and Machine Operators and Assemblers
- Other: ____________________

12. Total family income (gross) per month : *

Mark only one oval.

- ( ) < RM 4,000
- ( ) RM 4,000 - RM 9,000
- ( ) > RM 9,000

Section B.

13. During the last few months, have you experienced any of the following : *

Mark only one oval per row.

	Yes	No
Help or assistance from a stranger	( )	( )
Adverse discrimination from a stranger	( )	( )
Difficulties due to changes in your living conditions, including hostel disclosures	( )	( )
Difficulties in travelling	( )	( )

14. Relative to BEFORE COVID-19 crisis, how would you rank your CURRENT level of : *

Mark only one oval per row.

	N/A or I do not know	Much worse than before	Worse than before	Same as before	Better than before	Much better than before
Health care access	◯	◯	◯	◯	◯	◯
Internet access	◯	◯	◯	◯	◯	◯
Ability to pursue your studies, including your graduation and/or degree completion	◯	◯	◯	◯	◯	◯
Ability to socialize	◯	◯	◯	◯	◯	◯
Overall psychological well-being, including feelings of anxiety and/or depression	◯	◯	◯	◯	◯	◯

Section C.

*Note: Self-learning can be define as: Learning done by oneself, without a teacher or instructor.

15. Learning habits during COVID-19: *

Mark only one oval per row.

	Less than 2 hours	Between 2 hours to 4 hours	Between 4 hours to 6 hours	Between 6 hours to 8 hours	More than 8 hours
BEFORE COVID-19 crisis, how many hours do you spend per day for self-learning?	◯	◯	◯	◯	◯
DURING COVID-19 crisis, how many hours do you spend per day for self-learning?	◯	◯	◯	◯	◯
Regarding hours of learning DURING COVID-19 crisis, how many hours are online learning per day?	◯	◯	◯	◯	◯

16. I think that self-learning during COVID-19 is necessary because : *

Mark only one oval per row.

	Strongly disagree	Disagree	Neither agree nor disagree	Agree	Strongly agree
I can assure my learning progress.	⬭	⬭	⬭	⬭	⬭
I can maintain my learning habit.	⬭	⬭	⬭	⬭	⬭
My lecturers advise/inform me it is necessary and important.	⬭	⬭	⬭	⬭	⬭
My parents advise/inform me it is necessary and important.	⬭	⬭	⬭	⬭	⬭
My siblings advise/inform me it is necessary and important.	⬭	⬭	⬭	⬭	⬭
My friends advise/inform me it is necessary and important.	⬭	⬭	⬭	⬭	⬭

17. I consider my self-learning activities are effective because : *

Mark only one oval per row.

	Strongly disagree	Disagree	Neither agree nor disagree	Agree	Strongly agree
I have motivation for self-learning.	⬭	⬭	⬭	⬭	⬭
I have proper concentration skill.	⬭	⬭	⬭	⬭	⬭
I have support from my family.	⬭	⬭	⬭	⬭	⬭
I have an effective and conducive learning environment.	⬭	⬭	⬭	⬭	⬭
I can define my daily learning objectives.	⬭	⬭	⬭	⬭	⬭
I have various learning resources.	⬭	⬭	⬭	⬭	⬭
I communicate and collaborate with my friends about learning.	⬭	⬭	⬭	⬭	⬭

18. During COVID-19 crisis, I have learnt additional knowledge on : *

Mark only one oval per row.

	Strongly disagree	Disagree	Neither agree nor disagree	Agree	Strongly agree
Preventive health care.	⬭	⬭	⬭	⬭	⬭
Coronavirus.	⬭	⬭	⬭	⬭	⬭
Sustainable environment development.	⬭	⬭	⬭	⬭	⬭
E-learning tools and techniques.	⬭	⬭	⬭	⬭	⬭

Your cooperation and honesty for taking part in this survey are highly appreciated. Thank you.

This content is neither created nor endorsed by Google.

Google Forms
